# A study on the effects of the estrous cycle on uterine fluid and blood serum immunoglobulin G (IgG) content in the cow

**Published:** 2014

**Authors:** Sayed Mortaza Alavi-Shoushtari, Roya Abedizadeh, Amir Khaki, Aram Mokarizadeh, Kamran Dorostkar

**Affiliations:** 1 *Department of Clinical Sciences, Faculty of Veterinary Medicine, Urmia University, Urmia, Iran; *; 2 *Department of Immunology, Faculty of Medicine, Kurdistan University of Medical Sciences, Sanandaj, Iran; *; 3 *Department of Clinical Sciences, Faculty of Veterinary Medicine, Kurdistan University, Sanandaj, Iran.*

**Keywords:** Cow, Estrous cycle, Immunoglobulin G, Uterine fluid

## Abstract

To investigate the IgG content and its variations in uterine fluid (UF) during the estrous cycle of the cow and to compare them with those of the blood serum (S), six pairs of serum and UF samples for each phase of the cycle selected out of 240 bovine genital tracts and blood samples were collected from Urmia abattoir. The UF samples were collected by gentle scraping of the endometrium using a curette after uterine incision and their IgG content and those of the serum were measured by single radial immuno-diffusion (SRID) assay. Serum IgG values (Mean ± SEM) were generally higher than the UF values throughout the cycle except for di-estrus (S: 38.50 ± 0.90, UF: 51.60 ± 2.10 mg mL^-1^), in which the highest values were observed in UF samples. In met-estrus the difference was not significant (S: 34.80 ± 1.80 mg mL^-1^, UF: 30.80 ± 5.20 mg mL^-1^), however, in estrus the mean UF IgG value (12.50 ± 1.10 mg mL^-1^) was lower than that of the serum (31.30 ± 1.20 mg mL^-1^). In pro-estrus, the lowest values (S: 27.80 ± 1.30 mg mL^-1^, UF: 9.10 ± 1.50 mg mL^-1^) were obtained. The results showed a lower IgG values in the bovine UF than those of the serum in the follicular phase of the cycle, while in di-estrus the UF IgG content was the highest, suggesting some IgG production in the uterus at this phase.

## Introduction

Stage of the estrous cycle is an important determinant of resistance of the uterus to infection; with females of several species being more susceptible to infection during the luteal phase of the estrous cycle.^1^ Cyclic variations in resistance are likely caused by several mechanisms. For example, an increase in uterine motility during estrus when compared with di-estrus, could promote physical clearance of micro-organisms. Uterine synthesis and secretion of immunoglobulins also varies cyclically.1 In rats, secretion of immunoglobulin G (IgG) and immunoglobulin A (IgA) is maximal in pro-estrus when estrogen concentration is maximal,^2^ and plasma cells in the uterus increase in number during estrus in pigs.3 In horses, however, endometrial plasma cells tend to be more numerous during the luteal phase,^4^ as is true for the concentration of IgG and IgA in uterine flushings.^5^ Functional activity of polymorphonuclear leucocytes (PMNL) migration into the uterus could also be affected by the stage of the cycle.^1^

Progesterone is likely to play a major role in reducing uterine resistance to infection during the luteal phase in the cow. Ovariectomized cows treated with progesterone developed infection more frequently after experimental bacterial inoculation than cow treated with estrogens or untreated.^6^ In the presence of long-term elevations of the relatively immunosuppressive hormone, progesterone, chronic infections (typically endometritis and pyometra) can ensue.7 Estrogen may promote uterine resistance. Treatment of ovariectomized cows with estradiol cypionate increased phagocytic activity of uterine-derived PMNL,^1^ and lowered infection rate after intrauterine bacterial inoculation.^6^

Hussein *et al*. comparing different uterine immuno-globulin’s content with those of the serum in the sow concluded that IgG is the major immunoglobulin of uterine secretions,^8^ which is confirmed by other reports.^9^^,^^10^

Considering these reports, and to add more information to our previous report,^11^ this study was conducted to investigate cyclic changes of IgG content of the uterine secretions in pluriparous cows and to compare them with those of the blood serum.

## Materials and Methods

A total number of 240 genital tracts and blood samples were collected from mature local breed cows slaughtered in Urmia abattoir (northwest Iran, 45˚4´E and 37˚32´N). The reproductive stage of genital tracts was evaluated by considering structures existed on the ovaries and uterine tonicity according to the procedures described by Noakes *et al*.^12^ Those genital tracts considered being immature, anestrous, pregnant or abnormal were discarded and only those normal genital tracts regarded as being in pro-estrus, estrus, met-estrus or di-estrus phase of the cycle were used for this study. After inspection of the appearance of the uterine serosa to ascertain of being free of gross signs of abnormality, the uteri were separated from connective tissues, incised and the endometrium searched for any sign of abnormality. The samples with signs of inflammation, congestion and hemorrhage or pus were discarded. Then, the UF was collected by gentle scraping the endometrium using a curette, transferred in 2 mL Eppendorf tubes and stored at – 20 ˚C until examination. 

Blood samples were collected from jugular veins in plain test tubes, clotted at room temperature, sera harvested and kept frozen (– 20 ˚C) until examination. 

In this way six pairs of uterine fluid and serum samples in each phase of estrous cycle were selected for the study. 

Quantitative analysis of IgG content of uterine and serum samples obtained at different phases of the estrus cycle was performed by single radial immunodiffusion (SRID) assay according to a protocol described by Hay and Westwood with some modifications.^13 ^Briefly, 3% bovine anti-IgG antiserum (Sigma-Aldrich, St. Louis, USA) was added to final volume of 2% molten agar (Sigma-Aldrich, St. Louis, USA) prepared in a barbitone buffer in 56 ^°^C, after mixing, prepared molten agar was plated.

Following punching of wells, established wells were filled with samples and standard solutions prepared in five serial concentrations of 5, 10, 25, 50, 100 mg mL^-1 ^IgG, using a commercially available bovine IgG preparation (Sigma-Aldrich, St Louis, USA). After 24 hr incubation in a moist chamber in 37 ^°^C, rings diameters were measured using a standard curve depiction.

One-way ANOVA and Tukey’s test were used for the analysis of the data obtained from uterine and serum samples in different phases of the estrous cycle using SPSS (Version 16.0 for windows, SPSS Inc., Chicago, USA) software. Paired sample *t*-test was used to compare the serum and uterine fluid results and the significance was attributed as *p* < 0.05.

## Results

The results of IgG concentration estimations in the S and UF samples are depicted in Table 1. In pro-estrus, estrus and di-estrus the IgG content (Mean ± SEM) in UF samples (9.10 ± 1.50, 12.50 ± 1.10 and 51.60 ± 2.10 mg mL^-1^, respectively) were significantly different from those of the serum (27.80 ± 1.30, *p* < 0001; 31.30 ± 1.20, *p* < 0.001 and 38.50 ± 0.90, *p* = 0.004 mg mL^-1^, respectively). The mean UF IgG value in pro-estrus and estrus was significantly different from those of met-estrus and di-estrus, however, the difference between pro-estrus and estrus, and also, met-estrus and di-estrus was not significant, (*p* > 0.05). A significant difference was observed among the mean IgG value in S samples of pro-estrus, met-estrus and di-estrus, and also in samples of estrus and di-estrus, (*p* < 0.05). 

**Table 1:** Blood serum and uterine fluid IgG content during the different stages of the estrous cycle (Mean ± SEM).

**Stage **	**No.**	**Serum ** **(mg mL** ^-1^ **)**	**Uterine fluid ** **(mg mL** ^-1^ **)**	***p-*** **value**
**Pro-estrus**	6	27.80 ± 1.30 a	9.10 ± 1.50*^a^	0.000
**Estrus**	6	31.30 ±1.20 ac	12.50 ± 1.10*^a^	0.000
**Met-estrus**	6	34.80 ± 1.80 bc	30.80 ± 5.20^b^	0.479
**Di-estrus**	6	38.50 ± 0.90 b	51.60 ± 2.10*^c^	0.004

* indicates significant difference compared to blood serum.

abc different superscript letters indicate significant difference (*p* < 0.05) within columns.

## Discussion

The uterus and its luminal fluid components are of great importance in animal reproduction, and many attempts have been made to investigate its composition. Basna *et al*. cultured endometrial cells of the sow and analyzed the culture media;^14^ Dixon and Gibbons flushed the cow’s uterus with phosphate buffered saline;^15^ Roberts *et al*. used saline solution for uterine flushing in sheep, concentrated the obtained flushing, and measured its protein content.^16^ While, Troudson and Liu used absorbing sponges to obtain uterine secretions.^17^ These techniques give a diluted uterine fluid which can make the results doubtful. On the other hand, manipulation of the uterus during the luteal phase of the cycle is difficult to perform and may cause some damage to the genital tract.^18^^-^^20^ In this study undiluted uterine secretions were directly collected from the uterus and used for assays. Thomas has reviewed the plasma (not serum) proteins, including immunoglobulin, and their physiological significance.^21^ Murphy and Ballejo have reviewed the expression of growth factors and cytokines in the endometrium, and have discussed their biological role in the endometrium biology.^22^ Bondurant in a review of inflammation in the bovine female reproductive tract, points to the antigen-specific IgG_1_ and IgG_2_, but not IgA, and their serum and uterine origin in estrous uterine secretions.^9^ He also describes the events leading to IgG production and transfer to the uterine lumen, and its role in uterine resistance to pathogen organisms. At pro-estrus or estrus, the uterus may be exposed to sperm and seminal plasma antigens and to microbial antigens. The high E_2_ levels that prevail at this time may induce and increase in antigen-presenting efficiency in uterine cells. When rats were immunized in the intra-peritoneal space or intra-Payers’ patches, E_2 _appeared to enhance the secretion of specific antibodies of both IgA and IgG isotypes into the uterine lumen but lowered the amount secreted by vaginal mucosa.^9^

Reviewed the literature authors have mostly measured the IgG content of the serum and uterine fluid qualitatively and just in two phases as ‘luteal’ and ‘follicular’. Therefore, presenting the values for all the four stages of the estrous cycle in this work seems to be original.

In this study, the mean serum IgG values in luteal phase of the cycle, met-estrus (34.80 ± 1.80 mg mL^-1^) and di-estrus (38.50 ± 0.90 mg mL^-1^), were in agreement with the values of 36.40 ± 6.70 mg mL^-1 ^during the luteal phase of the cycle reported by Brenner *et al*. in the cow,^23^ and also, our result for pro-estrus (27.80 ± 1.30 mg mL^-1^) and estrus (31.30 ± 1.20 mg mL^-1^) were in agreement with the value of 28.30 ± 5.30 mg mL^-1 ^around the estrus in their report. They also measured total IgG content of each uterine horn separately and reported its range of variation as 30.00 to 115.00 mg mL^-1 ^in one horn and from 24.00 to 70.00 mg mL^-1 ^in the other horn during the luteal phase but essentially undetectable at estrus. Our obtained values were the results of both horn samples of the uterus.

In this study the mean values obtained in the uterine fluid samples in pro-estrus and estrus were significantly lower than those of the serum, which was in agreement with the observation of Brenner *et al*. who reported that a drop in serum and uterine IgG occurred concomitantly with the drop in peripheral serum progesterone, from 2.00 to 3.00 ng mL^-1 ^at the luteal phase to less than 0.50 ng mL^-1 ^around estrus.^23^

Hussein *et al*. using radial immunodiffusion technique for IgG assay in the sow, found no difference between UF IgG content in di-estrus (0.32 mg mL^-1^) and estrus (0.34 mg mL^-1^).^8^ Widders *et al*. reported that in the mare uterus, IgG and IgA levels relative to total proteins were higher in estrogenic than in progestagenic secretions;^24^ the same observations were reported by Canning and Billington and Rachman *et al*. who used immuno-peroxidase staining technique to identify secreted IgG and IgA producing cells in the uteri of mice during the estrous cycle.^25^^,^^26^

Here, in met-estrus, we observed the mean IgG content of uterine fluid was nearly similar to that of the serum, which means that IgG transferred from the local uterine capillaries to the uterine lumen. But in di-estrus, the IgG content of the uterine fluid was significantly higher than that of the serum. This means that in di-estrus a portion of IgG is synthesized in the endometrium locally, while in other phases of the cycle the remainder is drawn from the local uterine blood supply, as reported by Dhaliwal *et al*. in the cow and Hussein *et al*. in the sow.^8^^,^^27^ The IgG content of the uterine fluid in pro-estrus and estrus (follicular phase of the cycle) was lower than that of met-estrus and di-estrus, while in di-estrus being the highest. This is in agreement with the report of Gallichan and Rosenthal in mice and rat,^28^ and is contrary to the statement of higher IgG in human cervical mucus of the pro-estrous,^29^ and in the uterus of the rat.^30^

The result of IgG estimations in this study in pro-estrus is in agreement with our previous report in which higher γ_1_ and γ_2 _globulin in the serum were observed than that of the uterine fluid,^11^ and contrary to this observation of lower values in di-estrus than that of the serum. In other stages of the cycle, however, the γ globulin content of the serum was higher than that of the uterine fluid but the difference was not statistically significant. 

In the present study, the uterine fluid IgG content in met-estrus, which is coincident with the time of ovulation in the cow, has increased significantly from low levels in pro-estrus and estrus and continues through di-estrus. This could be the reason for the difference in the time of IgG changes in the bovine and rodents, mare and humans because ovulation in cows takes place in met-estrus rather than estrus as in other species, and cattle is exceptional from this point of view.

Although the number of samples is small, the results may suggest that bovine cyclic changes in serum and uterine fluid IgG content are part of the defense mechanisms against pathogens and are under the influence of ovarian steroid hormones concentrations, and follow a pattern different from those reported in mice and rats.
